# Dynamics modeling of multicomponent metal ions’ removal onto low-cost buckwheat hulls

**DOI:** 10.1007/s11356-020-09864-0

**Published:** 2020-07-13

**Authors:** Elwira Tomczak, Wladyslaw Kaminski

**Affiliations:** Faculty of Process and Environmental Engineering, Lodz University of Technology, Wolczanska 213/215, 90-924 Lodz, Poland

**Keywords:** Potentially toxic metals, Sorption kinetics, Fixed-bed model, Water treatment

## Abstract

The process of adsorption from water solutions containing a ternary system of Cu (II), Zn (II), and Ni (II) ions onto buckwheat hulls as a biosorbent was considered. The sorption capacity for buckwheat hulls was determined in sorption equilibrium batch experiments. The sorption kinetics equation corresponding to the mechanism of metal ions with the adsorbent was assumed. A new method for modeling sorption in a packed column was presented. A system of partial differential equations describing the mass balance, due to the assumption of a properly defined variable, was transformed into a system of ordinary nonlinear equations, which enables the identification of object parameters. The sorption capacity of the sorbent, sorption isotherms, and kinetics equations were used in dynamics modeling.

## Introduction

The presence of heavy metals in water solutions is of great environmental concern. Metal ions, such as copper, zinc, and nickel, have a significant impact on the environment because of their toxicity and their tendency to accumulate in living organisms. They are often detected in surface waters and, above all, in industrial wastewater. Therefore, more attention is given to the development of modern technologies, which could reduce the contamination of heavy metals to an acceptable level. The removal of potentially toxic metal ions can be accomplished by various methods.

Among the various available methods, such as chemical precipitation, coagulation, membrane techniques, and ion exchange, adsorption offers the most promising results, when economic reasons and efficiency are taken into consideration. Recently, extensive research has been carried involving experiments on various materials, which can be applied to remove heavy metals from water using this method. Activated carbon, zeolites, and ion exchange resins are the most frequently used sorbents (Nejadshafiee and Islami 2020; Ghafar et al. 2020; Natalea et al. 2020; Lee et al. 2020).

Activated carbon is one of the oldest and widely used adsorbent for water and wastewater treatment when removing different pollutants (Demirbas 2009; Petrova et al. 2010; Ghosh et al. 2020). It is a universal adsorbent with a well-developed internal surface. Current research focuses on new technologies based on thermally and chemically modified activated carbon obtained from sewage sludge and agricultural and forest waste (Imamoglu and Tekir 2008; Kim et al. 2008; Krishnan et al. 2011; Bhatnagar et al. 2013; Li et al. 2019; Lata et al. 2019; Li et al. 2020; Shen et al. 2020).

Application of solid resins was the top technology for the purification and separation of metal ions from different aqueous solutions by means of adsorption (Sari et al. 2007; Hu et al. 2010; Wang and Peng 2010; Li et al. 2020; Uloa et al. 2020).

Natural zeolites are a significant group of low-cost sorbents. Zeolites occur naturally as minerals and are obtained by mining. In the paper (Nguyen et al. 2010; Wang and Peng 2010; Puspitasari et al. 2018), the application of natural or modified zeolites for water and wastewater treatment is discussed.

Chitin and chitosan, along with its derivatives, are a universal sorption material. Their application for the sorption of various water contaminants has been discussed on numerous occasions. Reports focus on the sorption of heavy metals, selected dyes, and other aromatic chemical compounds ( Guibal et al. 2005; Bhatnagar and Sillanpää 2009; Malamis and Katsou 2013; Jozwiak et al. 2018). Chitin and chitosan are widely used because of their highly developed microporous structure, high porosity, high exchange capacity, and universality in binding water contaminants.

Despite numerous advantages, popular sorbents also have disadvantages. They are relatively expensive and thus need to be regenerated to be reused in the process. Studies on adsorption focus on the search for natural waste products, industrial by-products, natural plant sorbents, etc. (Repo et al. 2010; Ghosh et al. 2020; Alalwan et al. 2020; Shaikh et al. 2018). These materials are readily available and cheap to process and have the desired sorption capacity, and as a result, they are qualified as low-cost adsorbents. These materials do not need to be regenerated at the end of the adsorption cycle and may be disposed of in a traditional way, i.e., through composting, combustion, and storage. They can also be regenerated if desorption of the adsorbed substance is technologically simple and inexpensive.

In the last decade, a growing interest in inexpensive adsorbents to remove heavy metal ions, dyes, and other substances from water has been observed. Novel studies are designed to investigate unconventional plant/natural lignocellulosic products as adsorbents, such as corn cobs, banana fibers, sawdust, buckwheat and rice hull, and peat. Biosorption has emerged as a promising technique for metal ions removal (Ali 2010; Ho et al. 2011; Saurabh and Abhijit 2017; Huang et al. 2020).

Basic research on adsorption covers several issues. The authors propose experiments and calculations using recommended classic mathematical models and their own approach.

The first step in the research is to study adsorption equilibrium. The resulting information helps to assess the sorption capacity of the sorbent. The next step is to study the kinetics of sorption, which allows for the determination of the sorption mechanisms. Finally, the research covers the study of sorption in the column called the dynamics of sorption. In this paper, equilibrium, kinetics, and dynamics of sorption will be evaluated on the basis of our own experiments.

The authors propose a universal approach complete with an experimental procedure, which describes the operation of the adsorption columns during the start-up and continuous work until breakthrough to obtain concentration profiles prior to column regeneration.

The proposed procedure consists of the following steps:

1. Measurements of equilibrium and kinetics in batch experiments—to determine the sorption capacity and process kinetics for a given system, e.g., plant sorbent – heavy metal ions. If experiments do not confirm a given sorbent’s suitability, the procedure is discontinued.
2. Selection of models for mathematical descriptions of equilibrium—based on literature reports or our own research. Information about the sorption mechanism (physical, chemical, or mixed) and a suitable equation should be used.
3. Modeling sorption isotherm, e.g., using the Langmuir or Freundlich isotherm or other approximation. Sorption capacity is determined in this way.
4. Description of process kinetics, e.g., using pseudo-first-order or pseudo-second-order equations.
5. Carrying out experiments in a laboratory packed column at different flow rates, bed heights, and initial solution concentrations—this leads to the application and solving of the selected mathematical models to calculate the concentration of the solution at the column outlet and the concentration of the adsorbed substance in the adsorbent, as well as the breakthrough curves for different process conditions.

## Materials and preparation methods

Reagents used for the experiments discussed in this paper were acquired from Fluka, Germany. Adsorbate solution was prepared using demineralized water, sodium hydroxide, and a suitable metal salt (CuSO_4_ × 5H_2_O, NiSO_4_ × 6H_2_O, ZnSO_4_ × 7H_2_O).

Buckwheat hull as a sorbent was investigated in this paper. It is natural low-cost sorbent (Saka et al. 2012; Yin et al. 2013), readily available in Poland (price 110 €/tone). Buckwheat hulls are the dried outer shells of buckwheat seeds (25–36% of the seed weight). Both buckwheat (*Fagopyrum esculentum*) and tartary buckwheat (*Fagopyrum tataricum*) are annual honey-yielding plants. Buckwheat is cultivated in Russia, China, and Brazil and covers smaller areas in the USA, Canada, Germany, Italy, Slovenia, and Poland. The plant is the source of buckwheat honey, groats, straw, and husks. Buckwheat seed is rich in healthy nutrients. Dietary fiber fraction analysis showed that the highest amount of cellulose is found in the hulls (72%) and useless waste (68%) (Wang et al. 2013).

Buckwheat hulls acquired directly from the local mill were used in the tests. The material of 963 kg/m^3^ in density had a uniform grain size distribution of 3–4 mm. The sorption capacity of natural hulls after being washed in water at *T* = 90 °C and modification with 5% NaOH at 25 °C was studied. Because adsorption onto chemically preprocessed buckwheat hulls brought better results, this form of buckwheat hulls was used for the present studies.

Ten batches of aqueous solutions were prepared with specific analyte concentrations (10–200 mg/dm^3^) and uniform composition. In a multicomponent solution, the concentration of each component in the mixture was the same, which means that the concentration of each of the cations in the solution was 50 mg/dm^3^. After preparation, washing, and drying (105 °C, 3 h), the samples of buckwheat hulls (5 g) were placed into conical flasks, and 200 cm^3^ of test solution was added (pH = 5). The mixture was then mechanically shaken in a water bath until adsorption equilibrium was achieved (*T* = 25 °C). At intervals that were initially 15 min and then longer, samples were taken and analyzed for compound content up to 30 h. Metal concentrations were determined by IC (ICS-1000, IonPac AS5A, Dionex, San Jose, USA).

The experimental setup for sorption in a packed bed consisted of a glass column with a diameter of 3.45 cm and a length of 70 cm. The column was filled with dry mass sorbent (*m*). The bed height (*h*), void fraction of the bed (*ε*), and sorbent density (*ρ*_s_) were controlled. Before starting the sorption measurement, the bed was conditioned using redistilled water for 2 h. At time *t* = 0, an aqueous solution of heavy metal ions was pumped (volumetric flow rate *Q*) into the column from the bottom to the top of the bed.

Process operation parameters for buckwheat hulls—Cu (II) + Zn (II) + Ni (II**)** system (ternary solution)—to verify the model presented in this paper were as follows: *m* = 77.0 g, 53.1 g, and 31.2 g, corresponding to *h* = 0.58 m, 0.40 m, and 0.235 m, respectively; *ρ*_s_ = 963 kg/m^3^; *ε* = 0.85; *c*_0_ = 20 mg/dm^3^, 35 mg/dm^3^, and 50 mg/dm^3^; and *Q* = 1 dm^3^/h, 2 dm^3^/h, and 3 dm^3^/h.

## Results and discussions

### Mechanism of sorption onto cellulose sorbent (buckwheat hulls)

With regard to its chemical composition, the hulls contain a cellulose-lignin complex as its main component (responsible for its sorption properties), tannins, and phenolic compounds (inhibiting the growth of microorganisms, Gram-negative and Gram-positive bacteria). In their paper, discussing the sorption properties of materials containing food fibers (Zemnukhova et al. 2005), interesting results for buckwheat are reported, i.e., enhanced sorption capacity for selected ions after pre-processing (thermal and chemical treatment described in Section 2) when compared with the raw material. Analysis of static exchange capacity (SEC) based on the cellulose skeleton structure suggests the presence of proton groups of the alcohol type. They are responsible for ion exchange and complexing alkali, transition groups, and most likely, metal cations (Stavitskaya et al. 2001). SEC analysis performed for the raw buckwheat hulls.

Adsorption onto plant cellulose materials is mixed in nature. If the material is preconditioned, it has a porous surface, which facilitates physical adsorption. If cellulose material has reactive groups, the chemical reaction of complexing/chelating may occur. Upon proper modification, adsorption may be based on ion exchange. Metal ion binding onto cellulose sorbents is dependent on several factors, such as the charge value, the nature of donor atoms in the ligands, and the sorbent structure, which is related to the modification type or the extent of cross-linking. Adsorption capacity may be changed through chemical modification, mainly because of the presence or introduction of cations, such as Na^+^, NH_4_^+^, or Ca^2+^, into the biopolymer structure during the modification process (which enhances the material’s adsorption capacity) (Stavitskaya et al. 2001; Zaidi et al. 2018).

**Fig. 1:**
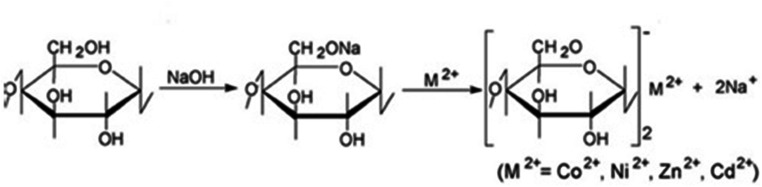
Ion exchange on cellulose sorbent after alkali modification

During alkali treatment, the replacement of labile hydrogen with sodium ion occurs. The sodium ion is more reactive than metal cations, such as Cu^2+^, Ni^2+^, Zn^2+^, Cd^2+^, and Co^2+^, which are present in the solution and can be easily exchanged. Typical ion exchange that occurs on cellulose materials is presented in Fig. 1.

The adsorbent surface is negatively charged. Increase in electrostatic attraction among cations enhances adsorption. A correlation was confirmed between the number of available functional groups and the amount of adsorbed metal. The pH of the sorbed solution is also important. In a solution with a lower pH, the adsorbent surface attracts more H^+^, thus reducing the attraction of metal ions, because there are more H^+^ ions, which compete with metal ions. At a higher pH, the anionic formations of hydroxide complexes decrease the concentration of free metal ions, and adsorption decreases. It has been shown that sorption decreases at low and high pH values in cellulose sorbents. The highest adsorption values were obtained at pH ≈ 5–7 (Stavitskaya et al. 2001).

### Modeling of sorption equilibrium

Mathematical modeling of sorption equilibrium is useful for analyzing and designing adsorption systems. The determination of sorbent-sorbate equilibrium in aqueous environments at constant temperature, i.e., the so-called sorption isotherm, is the basic element of the studies. The results of such experiments are equilibrium dependencies between the concentration of the sorbate in the sorbent and the concentration of the sorbate in the solution. Based on experimental data, with a known initial concentration *c*_0_ and equilibrium concentration *c*_e_, the sorption capacity *q*_e_ was determined for the solution using the following formula:

1 $$ {q}_{\mathrm{e}}=\frac{V}{m}\left({c}_0-{c}_{\mathrm{e}}\right) $$

where *c*_0_ and *c*_e_ are the initial and equilibrium concentrations of heavy metal ions in a solution [mg/dm^3^], *q*_e_ represents the equilibrium concentration of heavy metal ions in an adsorbent [mg/g], *V* represents the solution volume [dm^3^], and *m* represents the adsorbent mass [g d.m.].

To determine mathematical dependency between these values, typical relationships (two-parameter adsorption isotherm equations) are used as follows:

- Freundlich

2 $$ {q}_e={K}_{\mathrm{F}}{c_{\mathrm{e}}}^{1/{n}_{\mathrm{F}}} $$

- Langmuir

3 $$ {q}_{\mathrm{e}}=\frac{q_{\mathrm{m}}{K}_{\mathrm{L}}{c}_{\mathrm{e}}}{1+{K}_{\mathrm{L}}{c}_{\mathrm{e}}} $$

or three-parameter isotherm equations:

- Redlich-Peterson

4 $$ {q}_{\mathrm{e}}=\frac{q_{\mathrm{m}}{K}_{\mathrm{RP}}\kern0.43em {c}_{\mathrm{e}}}{1+{K}_{\mathrm{RP}}{c_{\mathrm{e}}}^{\mathrm{n}}} $$

- Radke-Prausnitz

5 $$ {q}_{\mathrm{e}}=\frac{K_{\mathrm{Rp}}{c}_{\mathrm{e}}}{1+A\cdot {c_{\mathrm{e}}}^{1-n}} $$

where *q*_m_ represents the adsorption capacity and *K*_L_ [dm^3^/g], *K*_F_ [dm^3^/g], *K*_RP_ [mg/g], *K*_Rp_ [mg/g], and *A* represent constants for the respective equations.

However, the approximations presented above were unsatisfactory, which was similar to the description of sorption in the papers (Kaminski et al. 2008; Li et al. 2008).

In this paper, it was found that a better approximation can be obtained if the exponential equation of the general form is used:

6 $$ {q}_{\mathrm{e}}=-A\exp \left(-\frac{c_{\mathrm{e}}}{B}\right)+A $$

where *A* and *B* represent constants.

**Fig. 2:**
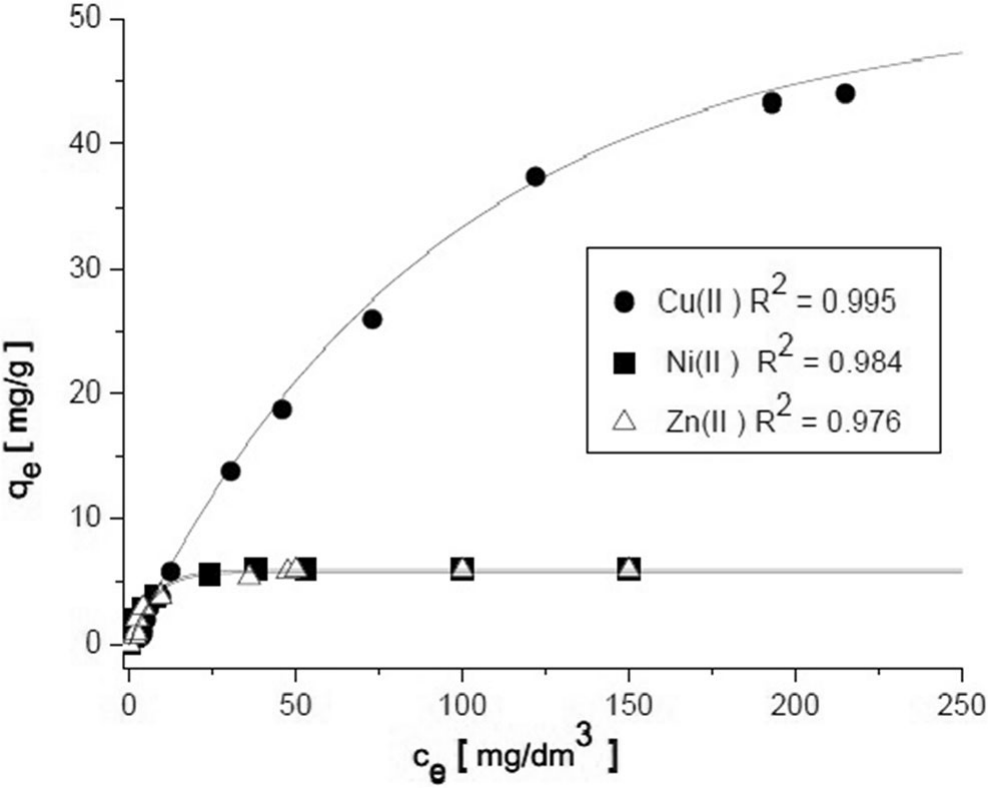
Sorption isotherms *q*_e_ = *f* (*c*_e_) for buckwheat hulls-multicomponent ion system (after preprocessing, pH = 5, *T* = 25 °C)

An equation describing the sorption isotherm has a horizontal asymptote, which is consistent with experimental data. Additionally, we would like to have consistent equations for all three solution components Cu (II), Ni (II), and Zn (II). Sorption isotherms for buckwheat hulls—the Cu (II) + Ni (II) + Zn (II) multicomponent system—are provided in Fig. 2. Maximum sorption capacities were determined—50 mg/g for Cu (II), 5.9 mg/g for Ni (II), and 5.6 mg/g for Zn (II). Table 1 presents approximating Eqs. (7), (8), and (9).

**Table 1:** Equations describing adsorption equilibrium

Sorbent	Adsorbate	Equation	*R*^2^ [-]	RMSE [mg/g]	Eq.
Buckwheat hulls	Cu (II)	*q*_e_ = − 50.714 exp(−*c*_e_/93.335) + 50.714	0.995	0.983	(7)
	Ni (II)	*q*_e_ = − 5.913 exp(−*c*_e_/6.912) + 5.913	0.984	0.264	(8)
	Zn (II)	*q*_e_ = − 5.716 exp(−*c*_e_/7.127) + 5.716	0.976	0.325	(9)

### Modeling of sorption kinetics

In the next step, sorption kinetics was determined. There are several widely used models of adsorption kinetics applied to study the mechanisms controlling the process, particularly chemical reaction and/or diffusion. Coefficients in kinetics models are obtained as a result of certain procedures, namely, linear and/or nonlinear regression analysis.

If adsorption is physical in nature and preceded by diffusion through the boundary layer, kinetics is most frequently described by the Lagergren pseudo-first-order equation (Lagergren 1898):

10 $$ \frac{dq}{dt}={k}_1\left({q}_{\mathrm{e}}-q\right) $$

Assuming that adsorption is controlled by chemisorption, pseudo-second-order model is described by the following expression (Blanchard et al. 1984; Ho 2006):

11 $$ \frac{dq}{dt}={k}_2{\left({q}_{\mathrm{e}}-q\right)}^2 $$

The two models are utilized quite frequently (Ho 2006; Ho et al. 2011). Experimental data are well described by the Lagergren equation only in the initial phase of the process. If the pseudo-second-order model describes experimental data well over the entire range, it can be stated that adsorption is based on the chemisorption mechanism.

The Elovich model is the appropriate model when chemisorption or mixed adsorption is the controlling process (Tomczak et al. 2015) and is expressed as follows:

12 $$ \frac{dq}{dt}={k}_{\mathrm{E}}{e}^{-\upbeta \times \mathrm{q}} $$

where *k*_1_ [1/min], *k*_2_ [g/(mg min)], and *k*_E_ [mg/(g min)] represent kinetics coefficients.

It was concluded that adsorption in a pure form, i.e., only physical or only chemical in character, occurs very rarely for most adsorbent-adsorbate systems. Most often, the process is more complex and complicated. Studies on adsorption include the search for new mathematical solutions that would describe the process well. Many papers and books on fractional calculus have recently appeared. They focus mainly on applications related to acoustic models, rheology, mechanical systems, object identification and control, robotics, etc. They can also be used in adsorption-related kinetics calculations. The authors’ own approach was presented in the paper (Tomczak et al. 2013) on metal ion sorption onto plant sorbents. Therefore, adsorption kinetics can be described by the three-parameter equation, allowing for *k*, kinetics constant; *α*, order of derivative; and order of *n*, kinetics equation of the general form (with initial condition *q*(0) = 0):

13 $$ \frac{d^{\upalpha}q}{d{t}^{\upalpha}}=k{\left({q}_{\mathrm{e}}-q\right)}^{\mathrm{n}} $$

Most fractional equations are devoted to the solvability of linear fractional equations in terms of special functions. Derivatives of integer order *α d*^α^ *f*/*dt*^α^ of function *f*(*t*) are classical and well defined. The fractional order of differentiation, α∈ < 0, 1 > is defined by the standard Riemann Liouville fractional derivative.

### Modeling of dynamics for buckwheat hulls

The main goal of this study was to develop a simple method for modeling the adsorption process in a packed column. It is an original approach to solving the mass balance in the packed column analytically. A mathematical model to calculate the concentration of solution at the column outlet and the concentration of adsorbed substance in the adsorbent, as well as breakthrough curves for different process conditions and column dimensions, was proposed in the study.

The assumption is that the description of sorption kinetics in the bed and the definition of variables follow the process from Lagrange’s point of view (moving observer), which allows for the calculation of the concentration in the solution and the sorbent as a function of time and distance from the inlet of the column. Assumptions of the model were presented in (Tomczak 2013).

Changes of concentration in the fluid *c*(*t*,*x*) and adsorbent *q*(*t*,*x*) are functions of time and distance from the inlet. Mass balance between fluid flow and the bed is achieved under the following conditions:

- Initial *c*(0,*x*) = 0

$$ q\left(0,x\right)=0, $$

- Boundary

$$ c\left(t,0\right)={c}_0\ \mathrm{for}\ t>0\ \left(\mathrm{constant}\ {c}_0\ \mathrm{concentration}\ \mathrm{at}\ \mathrm{column}\ \mathrm{inlet}\right), $$

- Adsorption kinetics equation is the same at each column section.

Equation (14) represents a general mass balance with the assumption presented above. The equation is well known and often cited in the literature (Babu and Gupta 2005).

14 $$ {u}_0\frac{\partial c\left(x,t\right)}{\partial x}+\left(1-\varepsilon \right){\rho}_{\mathrm{s}}\frac{\partial q\left(x,t\right)}{\partial t}+\varepsilon \frac{\partial c\left(x,t\right)}{\partial t}=\varepsilon {D}_{\mathrm{eff}}\frac{\partial^2c\left(x,t\right)}{\partial {x}^2\left(x,t\right)} $$

where *u*_0_ represents the apparent linear velocity [m/min] and *D*_eff_ represents effective diffusivity [m^2^/min].

To facilitate the interpretation of results, the introduction of the following variable was suggested:

15 $$ {\xi}_0={u}_0\cdot t $$

16 $$ \xi =\left\{\begin{array}{c}{u}_0t-x\kern1em for\kern0.33em {u}_0t>x\kern0.66em \\ {}0\kern3.12em for\kern0.33em {u}_0t\le x\kern0.66em \end{array}\right. $$

where *x* represents the distance from the column inlet.

Partial derivatives of *x* and *t* are converted into derivatives of *ξ* yield eqs. (17)–(20):

17 $$ \frac{\partial c\left(x,t\right)}{\partial x}=-\frac{d c\left(\xi \right)}{d\xi} $$

18 $$ \frac{\partial c\left(x,t\right)}{\partial t}={u}_0\frac{d c\left(\xi \right)}{d\xi} $$

19 $$ \frac{\partial q\left(x,t\right)}{\partial t}={u}_0\frac{d q\left(\xi \right)}{d\xi} $$

20 $$ \frac{\partial^2q\left(x,t\right)}{\partial {x}^2}=\frac{d^2q\left(\xi \right)}{d{\xi}^2} $$

After the substitutions of Eqs. (17), (18), (19), and (20) and the appropriate transformation, Eq. (14) takes the following form:

21 $$ -\frac{d c}{d\xi}+{\rho}_{\mathrm{S}}\frac{d q}{d\xi}=\frac{\varepsilon {D}_{\mathrm{eff}}}{u_0\left(1-\varepsilon \right)}\frac{d^2c}{d{\xi}^2} $$

In the paper, it was assumed that concentration changes in the sorbent are in line with the adsorption kinetics equation suitable for a given adsorbate-adsorbent system. If adsorption kinetics can be described by Eqs. (10), (11), (12), and (13) at any point in the column, then it is possible to find *q* = *f*(*ξ*).

The calculations were carried out in a MATLAB computing environment to verify the model using evolutionary algorithms as a mathematical tool support (Tomczak 2011).

Due to the assumption of a properly defined variable, the system of partial differential equations describing the dynamics of the adsorption column (mass balance) and sorption kinetics was transformed into a system of ordinary nonlinear equations, which enables the identification of object parameters in simple experiments.

Sorption capacity *q*_e_ was obtained in separate sorption equilibrium experiments presented earlier. For heavy metal ions, the relationship *q*_e_ = *f*(*c*_0_) (where *c*_0_ = *c*_e_) is represented by Eqs. (7), (8), and (9).

After adopting the appropriate sorption kinetics equations, the analytical form of the solution of Eq. (14) can be obtained.

Two adsorption mechanisms, namely, chemical and mixed adsorption, were analyzed for buckwheat hulls. In the first case, the model involving a pseudo-second-order kinetics equation was considered, and the second model comprised the Elovich equation.

When the assumption that chemical sorption occurring on buckwheat hulls can be adequately described using a form of the pseudo-second-order kinetics equation, the solution leads to the following equations:

22 $$ q\left(\xi \right)=\left\{\begin{array}{c}{q}_{\mathrm{e}}\frac{\alpha_2\xi }{1+{\alpha}_2\xi}\kern3em x<{u}_0t\\ {}0\kern7em x\ge {u}_0t\end{array}\right.\kern1em $$

23 $$ \left\{\begin{array}{c}c={c}_0+{\rho}_S{q}_{\mathrm{e}}\left(\frac{\alpha_2\xi }{1+{\alpha}_2\xi }-\frac{\alpha_2{\xi}_0}{1+{\alpha}_2{\xi}_0}\right)-\frac{\varepsilon {D}_{\mathrm{e}\mathrm{ff}}{q}_e{\alpha}_2{\rho}_S}{u_0\left(1-\varepsilon \right)}\left(\frac{1}{{\left(1+{\alpha}_2\xi \right)}^2}-\frac{1}{{\left(1+{\alpha}_2{\xi}_0\right)}^2}\right)\kern1em \\ {}\kern25.33em x<{u}_0t\\ {}c=0\kern23.66em x\ge {u}_0t\end{array}\right. $$

where

24 $$ {\alpha}_2=\frac{q_{\mathrm{e}}{k}_2}{u_0} $$

Detailed information on the model has been presented for the adsorption of heavy metal ions on chitosan beads in the paper (Tomczak 2013).

For mixed sorption, the Elovich equation in Eq. (12) (Chang and Juang 2005; Tomczak and Kaminski 2012) may by applied and defined as follows:

25 $$ \frac{\partial q}{\partial t}={u}_0\frac{d q}{d\xi}={k}_{\mathrm{E}}\exp \left(-\beta q\right) $$

Substituting Eq. (25) into Eq. (21) and the appropriate transformation results in the following formula:

26 $$ q\left(\xi \right)=\left\{\begin{array}{c}\frac{1}{\beta}\ln \left({\alpha}_{\mathrm{E}}\xi +1\right)\kern3em x<{u}_0t\kern1em \\ {}0\kern7.33em x\ge {u}_0t\end{array}\right.\kern2.00em $$

27 $$ \left\{\begin{array}{c}c={c}_0+\frac{\rho_{\mathrm{s}}}{\beta}\ln \left(\frac{\alpha_{\mathrm{E}}\xi +1}{\alpha_{\mathrm{E}}{\xi}_0+1}\right)-\frac{\varepsilon {D}_{\mathrm{eff}}{\alpha}_{\mathrm{E}}{\rho}_{\mathrm{s}}}{\beta {u}_0\left(1-\varepsilon \right)}\left(\frac{1}{\alpha_{\mathrm{E}}\xi +1}-\frac{1}{\alpha_{\mathrm{E}}{\xi}_0+1}\right)\\ {}\kern19em x<{u}_0t\\ {}c=0\kern17em x\ge {u}_0t\kern1em \end{array}\right.\kern1em $$

where

28 $$ {\alpha}_{\mathrm{E}}=\frac{k_{\mathrm{E}}\beta }{u_0} $$

In both analyzed cases, i.e., Eqs. (23) and (27), obtained results are presented in (Table 2). However, calculations were continued with Eq. (23), with the assumption that the adsorption kinetics in the column is mainly chemical in nature because the hulls were modified with NaOH solution (Na^+^ replacement with metal ion).

**Table 2:** Coefficients of the model identification and statistical evaluation for buckwheat hulls—Cu (II) + Ni (II) + Zn (II) system. Upper part is for the model with the pseudo second-order kinetics equation and lower one comprised the Elovich equation

*Q* (dm^3^/h)	*c*_0_ (mg/dm^3^)	*h* (m)	Ion	*k*_2_ (g/(mg min))	*q*_e_ (mg/g)	*D*_eff_ (m^2^/min)	*α*_2_ (1/m)	SUM (mg/dm^3^)	*R*^2^ (-)	*δ*_m_ (mg/dm^3^)
2.0	35	0.58	Cu (II)	2.68·10^−4^	15.134	7.12·10^−3^	0.114	56.199	0.883	2.260
			Ni (II)	4.42·10^−3^	5.866	2.95·10^−2^	0.726	24.314	0.990	1.487
			Zn (II)	6.29·10^−3^	5.605	4.89·10^−2^	0.988	14.132	0.994	1.133
*Q* (dm^3^/h)	*c*_0_ (mg/dm^3^)	*h* (m)	Ion	*k*_E_ (mg/(g min))	*β* (g/mg)	*D*_eff_ (m^2^/min)	*α*_E_ (1/m)	SUM (mg/dm^3^)	*R*^2^ (-)	*δ*_m_ (mg/dm^3^)
2.0	35	0.58	Cu (II)	0.666	0.486	7.12·10^−3^	9.081	89.178	0.815	2.848
			Ni (II)	0.419	2.3611	2.95·10^−2^	27.730	112.18	0.953	3.193
			Zn (II)	0.337	2.8272	4.89·10^−2^	26.736	86.039	0.965	2.797

Upper part is for the model with the pseudo-second-order kinetics equation and lower one comprised the Elovich equation

Calculations can be performed for each component of the multicomponent mixture. In this case, each component, e.g., heavy metal ion, will have an individual coefficient k and D_eff_” is proposed.

**Fig. 3:**
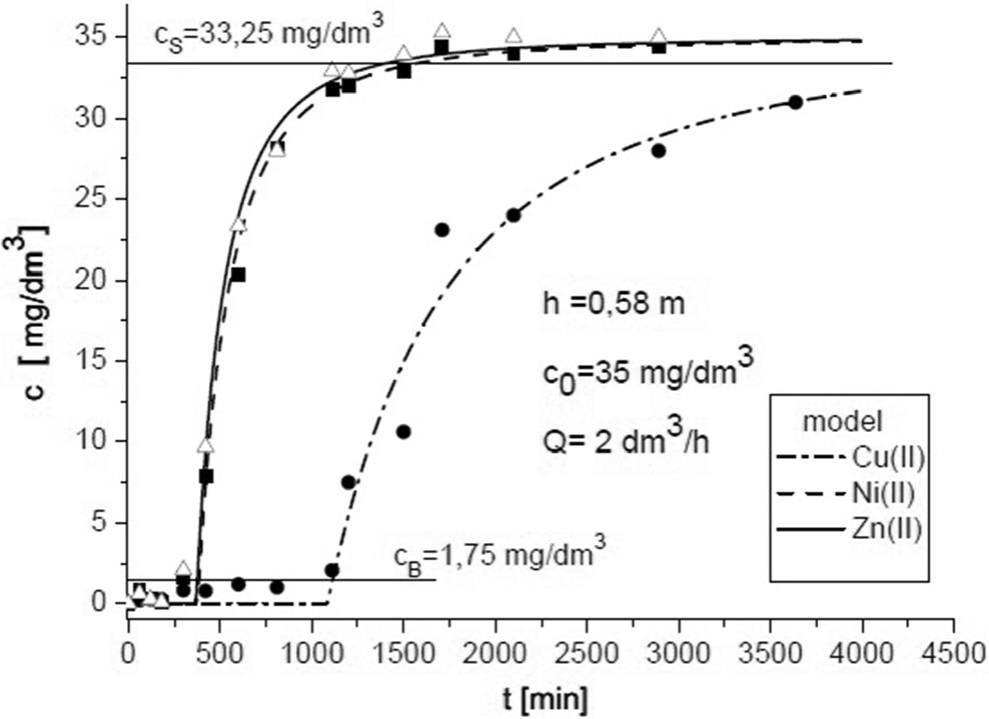
Concentration changes of Cu (II) + Ni (II) + Zn (II) in the solution at the column outlet

**Fig. 4:**
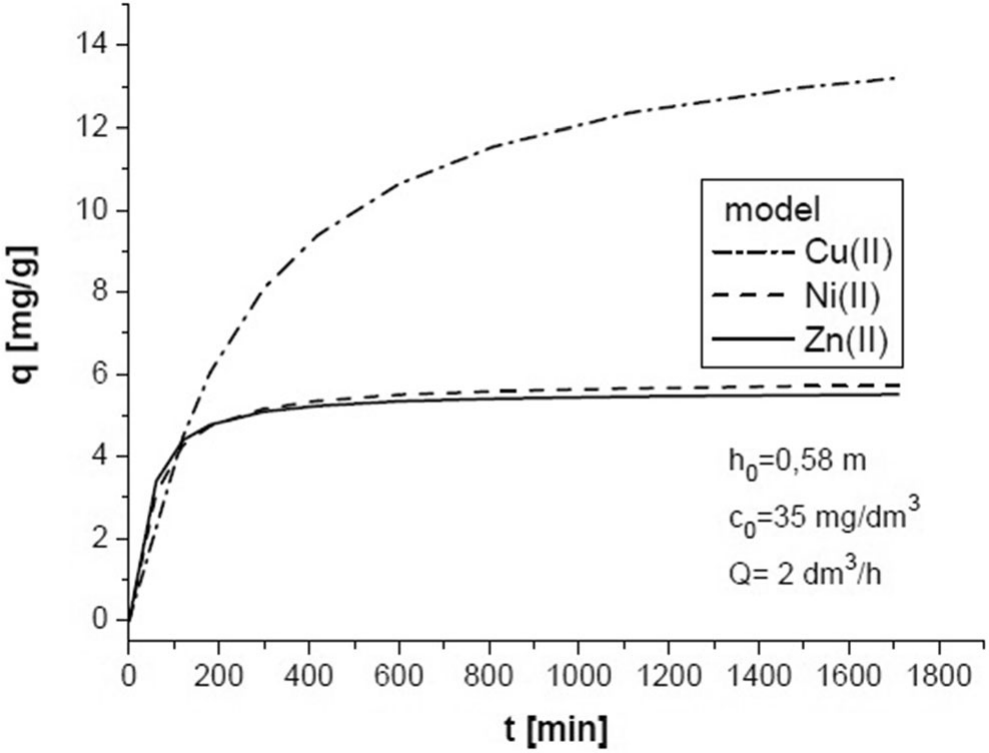
Concentration changes of Cu (II) + Ni (II) + Zn (II) in the sorbent at the column inlet

Figure 3 compares concentration *c* at the column outlet (experimental data for ternary ion mixture) with the values calculated using the model based on Eq. (23). The amount of adsorbed ions *q* using Eq. (22) is presented in Fig. 4 for selected process parameters. In this case, *q*_e_ was determined in equilibrium experiments and described by Eqs. (7), (8), and (9). The *q*_e_ determined for the concentration at the column inlet *c*_0_ = 35 mg/dm^3^ was as follows: Cu (II) = 15.134 mg/g, Ni (II) = 5.866 mg/g, and Zn (II) = 5.605 mg/g. Therefore, two remaining parameters in Eq. (23) were determined, i.e., *k*_2_ and *D*_eff_.

**Fig. 5:**
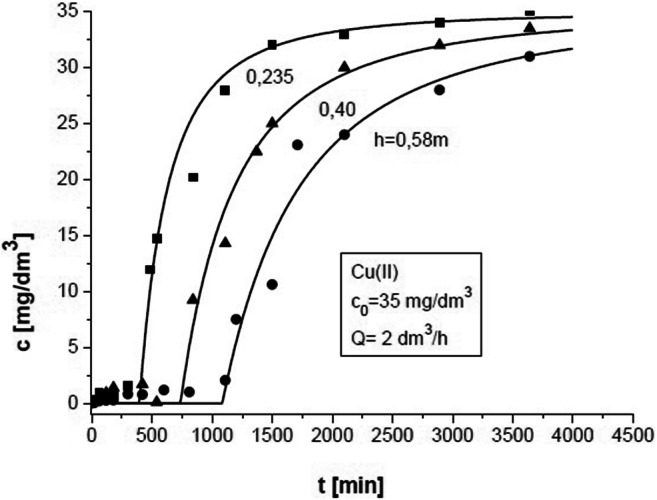
Concentration changes of Cu (II) in the solution at the column outlet depending on bed height

Experiments were conducted for different bed heights (*h* = 0.58 m, 0.40 m, and 0.235 m). They were used to verify model calculations. Example results for the adsorption of Cu (II) ions are presented in Fig. 5. Similar results were obtained for Ni (II) and Zn (II) ions.

The studies have demonstrated that calculations using the presented model yield satisfactory results. However, it must be underlined that buckwheat hulls show great preference for copper ions (15 mg/g d.m.), while other ions are sorbed over half as efficiently (approximately 6 mg/g d.m.).

Table 2 provides a list of coefficients identified in the model and a statistical evaluation of the calculations. In the statistical evaluation, the following factors were taken into account: the sum of squared deviations of the calculated and experimental values (SUM), the square of the determination coefficient (*R*^2^), and the root of mean square error (*δ*_m_).

The mathematical model applied and the identification of model parameters *k*_2_ and *D*_eff_ enabled (for the defined bed heights in the column) the calculation of the breakthrough time *t*_B_ corresponding to the outlet concentration equal to 0.05 *c*_0._

**Fig. 6:**
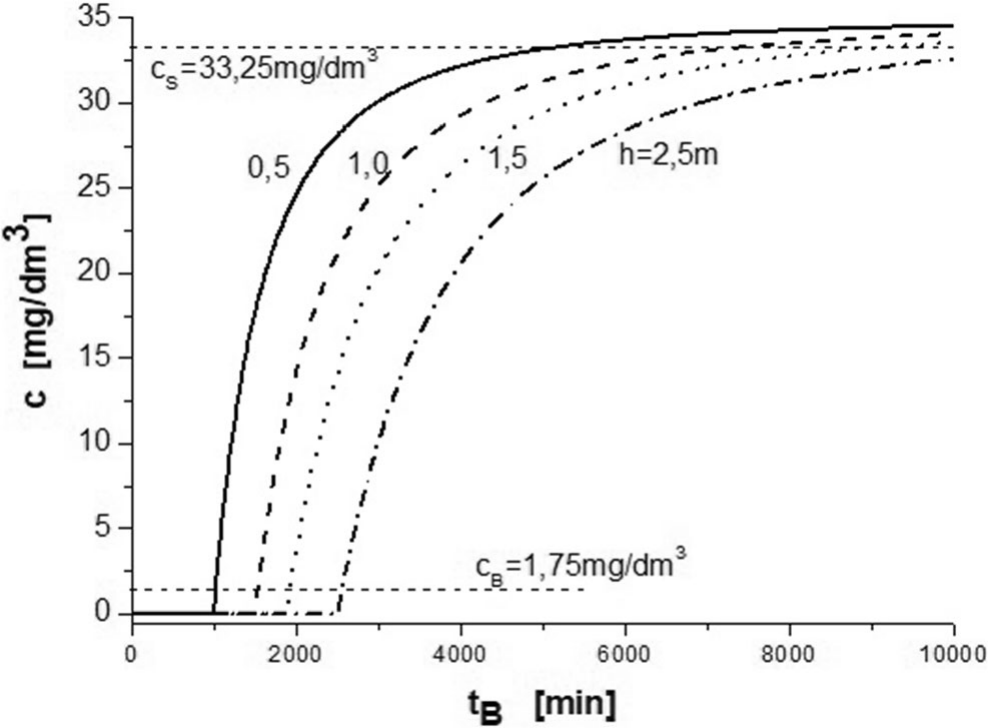
Concentration profiles for Cu (II) vs. time for *c*_0_ = 35 mg/dm^3^; *Q* = 2.0 dm^3^/h; and *h* = 0.5, 1.0, 1.5, and 2.5 m

Concentration profiles for Cu (II) vs. time were calculated for *c*_0_ = 35 mg/dm^3^; *Q* = 2.0 dm^3^/h (which corresponds to *u*_0_ = 3.5657·10^−2^ m/min); and column operation parameters at different bed heights, namely, *h* = 0.5, 1.0, 1.5, and 2.5 m, which are presented in Fig. 6. For *c*_0_ = 35 mg/dm^3^ at inlet to the column, the breakthrough and saturation concentrations are *c*_B_ = 1.75 mg/dm^3^ and *c*_S_ = 33.25 mg/dm^3^, respectively. For the bed heights in question, the breakthrough time is *t*_B_ = 1023, 1554, 1954, and 2575 min. Adequate calculations were performed for Zn (II) and Ni (II). The breakthrough time was much shorter compared with Cu (II).

## Conclusions

1. This research confirmed that after pre-treatment, buckwheat hulls can be used as a biosorbent for toxic heavy metal ion multicomponent system removal from water and wastewater.
2. The ability of buckwheat hulls to adsorb heavy metal ions in the following order, Cu (II) >> Ni (II) > Zn (II), was confirmed.
3. The model of process dynamics in the column presented in the paper took chemisorption kinetics (pseudo-second-order) into account.
4. The proposed variable transformation converts the system of partial differential equations into a system of ordinary equations, which enables an analytical solution to the system of equations.

## Nomenclature

*A*: Constant in Eq. (6) [mg/g]
*B*: Constant in Eq. (6) [mg/dm^3^]
*C*: Constant in Eq. (6) [mg/g]
*c*: Instantaneous concentration of heavy metal ions in a solution [mg/dm^3^]
*c*_0_: Initial concentration of heavy metal ions in a solution [mg/dm^3^]
*c*_B_: Breakthrough concentration [mg/dm^3^]
*c*_e_: Equilibrium concentration of heavy metal ions in an adsorbent [mg/dm^3^]
*c*_S_: Saturation concentration [mg/dm^3^]
*D*_eff_: Effective diffusivity [m^2^/min]
*h*: Bed height [m]
*k*_1_: Constant in pseudo-first-order equation [1/min]
*k*_2_: Constant pseudo-second-order equation [g/(mg min)]
*k*_E_: Constant Elovich equation [mg/(g min)]
*K*_F_: Constant in Freundlich equation [(mg g^−1^) (mg dm^−3^)^–1/nF^]
*K*_L_: Constant in Langmuir equation [dm^3^/g]
*K*_RP_: Constant in Redlich-Peterson equation [mg/g]
*K*_Rp_: Constant in Radke-Prausnitz equation [mg/g]
*m*: Adsorbent mass [g d.m.]
*n*: Kinetics order [-]
*1*/*n*_F_: Heterogeneity factor [-]
*q*_e_: Equilibrium concentration of heavy metal ions in an adsorbent [mg/g]
*q*_m_: Adsorption capacity [mg/g]
*Q*: Volumetric flow rate [dm^3^/h]
*R*^2^: The square of the determination coefficient [-]
RMSE: The root of mean square error [mg/g]
SUM: The sum of squared deviations of the calculated and experimental values [mg/dm^3^]
*t*: Time [min]
*t*_B_: Breakthrough time [min]
*u*_0_: The apparent linear velocity [m/min]
*V*: Solution volume [dm^3^]
*α*: Order of derivative [-]
*α*_E_: Variable defined by Eq. (28) [1/m]
*α*_2_: Variable defined by Eq. (24) [1/m]
*β*: Constant Elovich equation [g/mg]
*δ*_m_: The root of mean square error [mg/dm^3^]
*ε*: Void fraction of the bed [-]
*ζ*: Cumulative variable [m]
*ζ*_0_: Cumulative variable for *x* = 0 [m]
*ρ*_s_: Sorbent density [kg d.m./m^3^]
